# Follicular nodules (Thy3) of the thyroid: is total thyroidectomy the best option?

**DOI:** 10.1186/1471-2482-14-12

**Published:** 2014-03-06

**Authors:** Pietro Giorgio Calò, Fabio Medas, Rosa Santa Cruz, Francesco Podda, Enrico Erdas, Giuseppe Pisano, Angelo Nicolosi

**Affiliations:** 1 Department of Surgical Sciences, University of Cagliari, S.S. 554, Bivio Sestu, 09042 Monserrato, (CA), Italy

**Keywords:** Follicular neoplasm, Thyroid cancer, Thyroid, Fine needle aspiration, Cytology

## Abstract

**Background:**

Identification of the best management strategy for nodules with Thy3 cytology presents particular problems for clinicians. This study investigates the ability of clinical, cytological and sonographic data to predict malignancy in indeterminate nodules with the scope of determining the need for total thyroidectomy in these patients.

**Methods:**

The study population consisted of 249 cases presenting indeterminate nodules (Thy3): 198 females (79.5%) and 51 males (20.5%) with a mean age of 52.43 ± 13.68 years. All patients underwent total thyroidectomy.

**Results:**

Malignancy was diagnosed in 87/249 patients (34.9%); thyroiditis co-existed in 119/249 cases (47.79%) and was associated with cancer in 40 cases (40/87; 45.98%). Of the sonographic characteristics, only echogenicity and the presence of irregular margins were identified as being statistically significant predictors of malignancy. 52/162 benign lesions (32.1%) and 54/87 malignant were hypoechoic (62.07%); irregular margins were present in 13/162 benign lesions (8.02%), and in 60/87 malignant lesions (68.97%). None of the clinical or cytological features, on the other hand, including age, gender, nodule size, the presence of microcalcifications or type 3 vascularization, were significantly associated with malignancy.

**Conclusions:**

The rate of malignancy in cytologically indeterminate lesions was high in the present study sample compared to other reported rates, and in a significant number of cases Hashimoto’s thyroiditis was also detected. Thus, considering the fact that clinical and cytological features were found to be inaccurate predictors of malignancy, it is our opinion that surgery should always be recommended. Moreover, total thyroidectomy is advisable, being the most suitable procedure in cases of multiple lesions, hyperplastic nodular goiter, or thyroiditis; the high incidence of malignancy and the unreliability of intraoperative frozen section examination also support this preference for total over hemi-thyroidectomy.

## Background

Clinically palpable thyroid nodules are common and can be found in 4-20% of the adult population [1-4]. Ultrasound-detectable nodules, however, may be present in 30-50% of the population [3,5]. They are usually benign [1,2,6-8]; however, 5-15% prove to be malignant [1,2,4,5,7,9].

Fine needle aspiration cytology (FNAC) represents the main diagnostic tool currently used in the evaluation of thyroid nodules due to its high sensitivity, specificity, accuracy, reproducibility, and low cost [2-5,9-14]. A number of classification systems are used in the evaluation of FNAC results. In the guidelines published in 2006 by the American Thyroid Association (ATA) and the American Association of Clinical Endocrinologists (AACE), cytological findings that are suspicious for malignancy as well as follicular lesions and follicular neoplasms (FN) were classified under the heading of “indeterminate”. These guidelines were revised by ATA in 2009, and the “indeterminate” classification was redefined to differentiate between follicular and Hürthle cell neoplasms and follicular lesions of undetermined significance. Hürthle cell lesions have been described as a subtype of FN in the 2010 Bethesda FNAC classification system [5]. In 2007, the ‘Thy’ classification system was introduced by the British Thyroid Association (BTA) to guide the management of the thyroid nodules based on FNAC analysis of the thyroid. They suggested a management plan for each of the five diagnostic categories obtained (Thy1 to Thy5) [2].

Identification of the best management strategy for nodules with Thy3 cytology, which accounts for 5-30% of all patients with thyroid nodules [1,3,7,11,15], presents particular problems for clinicians. Most patients with cytologically indeterminate nodules are referred for thyroid surgery due to the fact that the presence of follicular thyroid malignancy cannot be ruled out, even though the majority are subsequently identified as having benign disease [1,3,12,16,17]. For these patients, thyroid surgery was unnecessary, yet it exposed them to a 2-10% risk of serious surgical complications, and patients are then required to take levothyroxine replacement therapy for life [1,12]. In the literature, the reported malignancy rate for indeterminate lesions (Thy3) varies between 3% and 52% of Thy3 cases [2,15,18-20].

Efforts have been made to identify criteria able to predict malignancy in Thy3 nodules. Some studies have identified the male gender, age, nodule size, shape, border characteristics, hypoechogenicity, and the presence of microcalcifications as risk factors for malignancy in indeterminate follicular lesions [3,4,21], but other studies show no evidence for this [14,22].

Here, we report the clinical, cytological (FNAC) and sonographic data obtained from 249 patients diagnosed with “follicular neoplasm” (Thy3) and correlate the data with the malignancy rates determined post-thyroidectomy with the scope of identifying risk factors of malignancy and to determine whether total thyroidectomy was indeed the best therapeutic option for these patients.

## Methods

Thyroid FNACs performed in patients referred to the Department of Surgery of the University of Cagliari (a tertiary care referral endocrine surgical center) between January 2009 and December 2012 were classified according to the guidelines published by the British Thyroid Association as: non diagnostic (Thy1), benign (Thy2), indeterminate (Thy3), suspicious for malignancy (Thy4), or malignant (Thy5). According to these evaluations, 249 cases with indeterminate nodules (Thy3) were included in this retrospective study. Of these, 198 were female (79.5%) and 51 male (20.5%), with a mean age of 52.43 ± 13.68 years. All patients with Thy3 nodules underwent total thyroidectomy (TT), as routinely practiced in this Institute; indeed intraoperative frozen section examination was not used as a means of diagnosing thyroid cancer and to determine the extent of thyroidectomy since our pathologists consider the usefulness of this procedure to be very limited with little benefit in terms of patient outcome. Age, gender, presence of thyroiditis, nodule size, and border characteristics were compared with definitive pathology. Ultrasonographic examinations and FNACs were performed with a 10 MHz linear transducer. The former technique was used to assess: thyroid nodule dimension, features of thyroid parenchyma, nodule structure, echogenicity, edge regularity, calcification pattern, and vascularization pattern. The color Doppler pattern of the thyroid nodules was evaluated and classified as follows: CDI (no vascularization), CDII (peripheral vascular signal), CDIII (central and peripheral vascular signal), and CDIV (diffuse vascular signal).

FNACs were performed with ultrasound guidance, using either a 22-gauge needle attached to a 10-mL disposable plastic syringe or an aspirator. Samples were stained with hematoxylin and eosin and evaluated by our Pathology Department. Only patients presenting Thy3 cytology were included in the study. Associations between the ultrasonographic features of the nodules and the histopathological results were evaluated to investigate whether nodule characteristics were predictive for malignancy.

The study has been performed in accordance with the Declaration of Helsinki. Ethical approval for our study was obtained from institutional ethical committee of Monserrato University Hospital, Cagliari. All patients provided written informed consent for their involvement in this study and for the storage and use of their data.

Surgical complications were assessed over a follow-up period that ranged between 6 and 54 months. Routine pre- and post-operative fibrolaryngoscopy were performed in all cases; vocal fold paresis was considered definitive (paralysis) when still present 6 months after surgery. Serum calcium (normal value = 2.09-2.54 mmol/L) and iPTH (intact parathyroid hormone) levels (normal value = 1.06-6.89 pmol/L) were assessed on postoperative day 1. An iPTH serum level < 1.06 pmol/L was used to determine postoperative hypoparathyroidism (considered definitive when present 6 months after surgery).

### Statistical analysis

Data were analyzed using descriptive statistics: Pearson’s chi-squared (exact) tests were used for categorical variables, and independent Student’s t tests were used for quantitative variables. Data were reported as the mean value ± standard error of the mean (SEM). All calculations were performed using the software package GraphPad Prism, Version 5.0 for Windows (GraphPad Software, San Diego, CA, USA). Values were considered as statistically significant for p ≤ 0.05.

## Results

### Cyto-histological correlation

Hyperplastic nodular goiter was diagnosed in 107/249 cases (42.97%) by pathological examination, follicular adenoma in 55/249 cases (22.1%), and malignancy in 87/249 patients (34.9%). In particular, papillary thyroid cancer was found in 67/87 patients (77%), with 32/67 belonging to the follicular variant subtype. Follicular thyroid cancer was found in 18/87 cases (20.7%), whereas Hürthle cell carcinoma lesions were found in 2 cases (2.3%).

### Clinical data and histological diagnosis

Thyroiditis co-existed in 119/249 patients (47.79%); it was associated with cancer in 40 patients (40/87; 45.98%) and with benign pathology in 79/162 patients (48.77%: in 52 cases with a hyperplastic nodular goiter and in 27 with a follicular adenoma). Cancer patients had a mean age of 51.50 ± 14.95 years, were predominantly female (F/M = 4.11/1), and had a mean nodule size of 18.68 ± 11.45 mm (range 6–47 mm; 8/87 malignant nodules were < 10 mm, 9.2%). Patients affected by benign disease had a mean age of 52.98 ± 12.96 years, were predominantly female (F/M = 3.76/1), and had a mean nodule size of 17.90 ± 9.11 mm (range 7–45 mm; 16/162 benign nodules were < 10 mm, 9.9%). None of these characteristics were statistically correlated with malignancy (see Table 1).

**Table 1:** Clinical and pathological data

	**Benign pathology (162/249)**	**Cancer (87/249)**	**p**
Age	52.98 ± 12.96	51.50 ± 14.95	0.19
Female/male ratio	3.76	4.11	0.91
Tumor Size (mm.)	17.90 ± 9.11 (range 7–45)	18.68 ± 11.45 (range 6–47)	0.27
Consensual thyroiditis	48.77%	45.98%	0.77

### Ultrasonographic findings

Microcalcifications were present in 34/162 benign lesions (20.99%) and in 25/87 malignant lesions (28.74%). Type 3 vascularization was present in 88/162 benign lesions (54.32%) and in 51/87 malignant lesions (58.62%); neither association was statistically significant. 52/162 benign lesions (32.1%) and 54/87 malignant were hypoechoic (62.07%). Irregular margins were present in 13/162 (8.02%) benign lesions and 60/87 malignant lesions (68.97%); these data were statistically significant (see Table 2). Hypoechoic nodules associated with irregular margins were present in 53/87 malignant lesions (60.9%) and in 10/162 benign lesions (6.17%) (p < 0.001).

**Table 2:** Ultrasonographic findings

**Variables**	**Benign pathology (162/249)**	**Cancer (87/249)**	**p**
*Echogenicity (%)*			
Hypoechoic	52/162 (32.1%)	54/87 (62.07%)	**< 0.001**
Isoechoic + Hyperechoic	110/162 (67.9%)	33/87 (37.93%)	
*Microcalcifications (%)*			
Presence	34/162 (20.99%)	25/87 (28.74%)	0.22
Absence	128/162 (79.01%)	62/87 (71.26%)	
*CD Vascularization (%)*			
CDIII vascularization	88/162 (54.32%)	51/87 (58.62%)	0.60
CD I + II vascularization	74/162 (45.68%)	36/87 (41.38%)	
*Margin irregularity (%)*			
Presence of irregular margins	13/162 (8.02%)	60/87 (68.97%)	**< 0.001**
Absence	149/162 (91.98%)	27/87 (31.03%)	

In boldface statistically significant data.

### Surgical treatment and outcomes

TT was performed in all patients (249/249). Prophylactic VI level lymphectomy was not performed in any patient; although this procedure is used in our Institute in cases of macroscopic suspect nodes, considering the great efficacy of radionuclide therapy and fewer associated complications.

Recurrent laryngeal nerve transient paresis occurred in 2/249 patients (0.8%) and permanent hypoparathyroidism occurred in 4/249 patients (1.6%). A cervical hematoma was reported in 3/249 patients (1.2%); of these, 2 required reintervention for bleeding control. Wound infection occurred in just a single patient (0.4%).

## Discussion

The increasing use of thyroid ultrasound scans has triggered an epidemic of thyroid nodules [3]. Thyroid FNAC is the most widely used diagnostic test in the management of such nodules [11,16]. However, the usefulness of FNAC is limited when dealing with follicular lesions presenting cellular atypia of indeterminate significance (Thy3), for which the distinction between benign and malignant lesions is ambiguous [11].

Indeterminate follicular lesions comprise 10% to 30% of cytopathological diagnoses [20]. This potentially places a large economic burden on the health care system due to the necessity for additional testing (FNAC and/or imaging) and may heighten patient anxiety [20]. Overall rates of malignancy in nodules cytologically diagnosed as FN have been reported to range between 16-52%, although the majority of studies report rates between 20-30% [2-5,7,8,11,15,19].

In the present retrospective study of 249 patients presenting indeterminate nodules (Thy3), 87 carcinomas were identified (34.9%) – one of the highest malignancy rates reported in the literature to date. This might be due to the criteria used by our cytopathologists for the diagnosis of follicular neoplasm when follicular cells are seen on aspirates. Another reason might be due to the fact that our institution is a tertiary care referral center; thus, patients presenting suspicious clinical or cytological features suggestive of malignancy may have been preferentially referred to our department, causing a bias in patient selection.

Papillary thyroid cancer was identified as the most common subtype of differentiated thyroid cancer diagnosed in atypical cells. This is consistent with other reports [22].

Several authors have attempted to classify a subset of nodules based on several clinical features and cytological characteristics with the aim of predicting malignancy in indeterminate nodules with greater accuracy [15]. The clinical features previously explored and suggested to be indicative of a malignant index nodule include: the male sex, a young age (< 40 years), large nodule size (> 30–40 mm), a high level of fixity to surrounding tissues, hypoechogenicity, and the presence of microcalcifications within nodules. Nevertheless, other authors have reported the relative lack of accuracy of such clinical parameters in predicting malignancy in indeterminate thyroid nodules [3,15,16,19,20,22].

The male sex has been reported to represent a risk factor for malignancy [3,21], but this finding has not been confirmed by others [8,14], nor in the present study. However, in the study performed by Sorrenti and colleagues [4], males had an increased risk of malignancy when the cytological diagnosis was Hürthle cell neoplasm.

The literature on the association between patient age and thyroid cancer is inconsistent. Some authors report no association between patient age at diagnosis and malignancy [4,5,8], while others have identified a higher risk of malignancy in younger patients with follicular neoplasms [14,21]. In contrast, two recent studies reported that patients above 40 or 50 years of age presented an increased incidence of malignancy [16,19]; while others simply report a trend towards a higher prevalence of malignancy with increasing age [8]. Other recent papers seem to provide an explanation to these contradicting results: by analyzing the distribution of cancer across different age decades, the risk of malignancy was found to increase at the two age extremes (< 30 yrs and > 60 yrs) [4,14,21]. In the present study, the mean age of patients was slightly lower in malignant lesions, but the data were not statistically significant; a similar result has also been reported in other studies [4].

Several authors have asserted that bigger nodule sizes are associated with malignancy [5], but others do not confirm this [8,19,21]. Here, overall nodule size did not result as a predictor of malignancy. Although the mean size of malignant nodules was slightly larger than non malignant ones, the difference between the groups was not statistically significance.

The relationship between Hashimoto’s thyroiditis and thyroid cancer is a debatable matter [4]. Moreover, it was recently reported that an increased rate of suspicious cytology in thyroid nodules is associated with the presence of positive thyroid auto-antibodies [23]. However, the histological features of Hashimoto’s thyroiditis have rarely been analyzed in studies evaluating the rate of malignancy in indeterminate cytology. Two reports suggest that there is no association between Hashimoto’s thyroiditis and malignancy [4,21], in line with the results of the present study.

The sonographic features of thyroid nodules have consistently failed to predict malignancy in FN with indeterminate cytology [13,14,22]. However, a hypoechoic appearance, the presence of microcalcifications (defined as < 2 mm hyperechoic spots), irregular or blurred margins, and intra-nodular vascularization are all taken as potential markers of malignancy, especially when present in combination [5,11,21]. The solid hypoechoic appearance of thyroid nodules is considered by some to be the most sensitive ultrasound parameter in predicting malignancy [5,21]. In our study, the presence of microcalcifications and type 3 vascularization were more likely to be associated with malignancy, but the correlation did not reach statistical significance; while a hypoechoic appearance and irregular margins were highly predictive of malignancy, confirming the results of Cantisani [11].

Quantitative ultrasound elastography is more accurate than conventional ultrasound in the diagnosis of thyroid nodules and it is useful for the pre-surgical selection of patients [5,8,11,24]. Malignant nodules have been consistently reported to exhibit significantly greater stiffness compared to benign lesions [5,11,24].

Cytological features, such as nuclear atypia, hypercellularity, lack of colloid, and the hypercellularity to colloid amount ratio, all increase the index of suspicion for malignancy; however, their predictive value is also widely variable [20]. Miller and colleagues [20] found that thyroid cancer was most common in patients with atypical cells. In the study by Dutta [15], only the absence of normal follicular cells in FNAC was predictive of malignant lesions, supporting the findings of previous reports [4,20]. In the present study, no significant correlations between cytological features and malignancy were revealed, probably because of the classification adopted.

At least 50 molecular markers have been analyzed in patients with thyroid nodules, and the use of immunocytochemistry for detecting abnormally high levels of thyroid peroxidase and galectin-3 in nodule FNAC seems to be particularly promising for predicting malignancy [15]. Nodule aspirates positive for: galectin-3, BRAF point mutations, the formation of PAX8-PPARγ1 chimeric oncogene, and the RET/PTC mutation have all been associated (even if to different extents) with thyroid cancer risk [8,12,17,25-27]; thus, the combined analysis of multiple markers is likely to be of the greatest clinical use in reducing the rate of unnecessary surgical procedures and associated complications [8]. Nacamulli [24] combined elastosonography with molecular testing for BRAF mutations and found that it improved the preoperative identification of thyroid malignancies; the author concluded that this strategy holds great potential for reducing the number of unnecessary surgical procedures. Alexander et al. [1] reported the results of a large, prospective, multicenter study validating a gene-expression classifier in patients with indeterminate thyroid nodules: 78 of the 85 nodules were identified as suspicious with a sensitivity of 92% and a specificity of 52%; the authors suggest that a more conservative approach should be considered for most patients with benign results from gene-expression classifier testing [1]. The clinical utility of molecular testing was also evaluated in the study by Nikiforov et al. [28], confirming a high positive predictive value of BRAF, PAX8/PPARγ, and RET/PTC mutations. However, in our opinion, although these preliminary studies are certainly promising, the molecular testing of FNAC samples is not yet suitable for standard practice.

No consensus has yet been reached in the literature regarding the management of patients with a diagnosis of Thy3 thyroid nodules resulting from FNAC analysis. A very important point of debate is the extent of surgery, implicating issues such as level of clinical risk as well as the economical costs associated with TT; various endocrinological society guidelines recommend surgery without specifying the extent of the treatment, yet no clinical investigation has confirmed survival or morbidity benefits associated with such a recommendation [8]. Several authors recommend TT as the first-line surgical approach for multinodular goiter, in particular for lesions greater than 1 cm in size, while others recommend loboisthmectomy for a solitary nodule [5,7,8,29]. The guidelines regarding simple lobectomy for Thy3 solitary nodules take into account nodule dimension and the availability of intraoperative examination of frozen sections for the assessment of malignancy [8]. However, the usefulness of intraoperative frozen section analysis is debatable, because the diagnosis of cancer depends on the demonstration of capsular and vascular invasion, which is often not identified in frozen sections [4,7,8]. This has indeed been the case in our experience and was the reason why frozen section histology was not performed. When a lobectomy is performed, patients must be informed about all risks including the possibility of a second operation being required, thus also the patient’s own wishes must be taken into account before commencing surgery [8].

In this study, the incidence of cancer was not negligible (34.9%) and in a significant number of cases the cancer was also associated with thyroiditis (45.98%). Hyperplastic nodular goiter was present in 42.97% of all patients and thyroiditis in 47.79%. These high rates are explained by the fact that Sardinia is an endemic area for thyroid diseases (hyperplastic goiter and Hashimoto’s thyroiditis in particular). Thus, considering the high incidence of cancer, the high rates of associated thyroid pathologies in Sardinia, and the current difficulties in preoperative and intraoperative diagnosis, we suggest that TT should be the treatment of choice for these patients. TT is a relatively safe treatment with few complications when performed by experienced endocrine surgeons; it also eliminates the risk of reoperation (following a positive diagnosis for malignancy after hemithyroidectomy, a relapse of goiter, or the development of a tumor in the residual lobe) and the related high incidence of complications [30-36].

## Conclusions

In conclusion, the risk of malignancy in cytologically indeterminate thyroid lesions was high in the present study population. Moreover, in a significant number of cases, Hashimoto’s thyroiditis and hyperplastic nodular goiter were also associated with the cancer.

Clinical and cytological features were all found to be inaccurate predictors of malignancy, and of the sonographic features only hypoechoic appearance and irregular margins were highly predictive of malignancy. Although various correlations between malignancy and certain parameters have been identified by other authors, the lack of any associations here may partly be explained by the relatively small sample size.

Thus, due to the relatively high risk of malignancy in indeterminate nodules observed here and the lack of highly accurate predictors, we recommend surgery as the first-line treatment of Thy3. Although, it is very difficult to standardize the treatment of follicular lesions, we prefer TT because it is the most suitable treatment for multiple lesions and when associated with hyperplastic nodular goiter and thyroiditis. Furthermore the high incidence of malignancy and the unreliability of intraoperative frozen section examination support TT as the preferred treatment in the large majority of cases. Nevertheless, hemithyroidectomy may be preferential in selected cases, such as single and small lesions, due to the low risk of associated complications.

Further multicenter studies could contribute to the standardization or modification of cytological classifications by identifying patients at high and low risk, thereby refining the recommended treatment profiles, in particular patient suitability for hemi- or total thyroidectomy. The wider use of immunocytochemical and genetic markers also holds promise for improving nodule classifications and identifying the patients with the greatest risk of developing thyroid cancer.

## Abbreviations

FNAC: Fine needle aspiration cytology; ATA: American Thyroid Association; AACE: American Association of Clinical Endocrinologists; FN: Follicular neoplasms; BTA: British Thyroid Association; TT: Total thyroidectomy; iPTH: Intact parathyroid hormone; SEM: Standard error of the mean.

## Competing interests

The authors declare that they have no competing interests.

## Authors’ contributions

PGC carried out part of the operations, conceived the study and drafted the manuscript. FM participated in the design of the study and performed the statistical analysis. RSC participated in the design of the study and in the acquisition of the data. FP revised the article and participated in the acquisition of the data. EE revised the article and participated in the acquisition of the data. GP carried out part of the operations and revised the article. AN participated in the design of the study and helped to draft the manuscript. All authors have read and approved the final manuscript.

## Pre-publication history

The pre-publication history for this paper can be accessed here:

http://www.biomedcentral.com/1471-2482/14/12/prepub
